# Radiotherapy refusal in breast cancer with breast-conserving surgery

**DOI:** 10.1186/s13014-023-02297-2

**Published:** 2023-08-05

**Authors:** Jiameng Liu, Zhanlin Zhu, Zhipeng Hua, Weijie Lin, Yiyin Weng, Juli Lin, Hehui Mao, Lifen Lin, Xuming Chen, Jujiang Guo

**Affiliations:** Department of Breast Surgery, Women and Children’s Hospital, School of Medicine, Xiamen University, No.10, Zhenhai Road, Xiamen, 361003 Fujian Province China

**Keywords:** Breast cancer, Radiotherapy, Breast-conserving surgery, Overall survival, Breast cancer specific survival

## Abstract

**Background:**

Although radiotherapy after breast-conserving surgery has been the standard treatment for breast cancer, some people still refuse to undergo radiotherapy. The aim of this study is to identify risk factors for refusal of radiotherapy after breast-conserving surgery.

**Methods:**

To investigate the trend of refusing radiotherapy after breast-conserving surgery in patients with breast cancer using the Surveillance, Epidemiology, and End Results database. The patients were divided into radiotherapy group and radiotherapy refusal group. Survival results were compared using a multivariate Cox risk model adjusted for clinicopathological variables. Multivariate logistic regression was used to analyze the influencing factors of patients refusing radiotherapy after breast-conserving surgery and a nomogram model was established.

**Results:**

The study included 87,100 women who underwent breast-conserving surgery for breast cancer between 2010 and 2015. There were 84,948 patients (97.5%) in the radiotherapy group and 2152 patients (2.5%) in the radiotherapy refusal group. The proportion of patients who refused radiotherapy after breast-conserving surgery increased from 2.1% in 2010 to 3.1% in 2015. The Kaplan–Meier survival curve showed that radiotherapy can improve overall survival (p < 0.001) and breast cancer specific survival (p < 0.001) in the patients with breast-conserving surgery. The results of multivariate logistic regression showed that age, income, marital status, race, grade, stage, subtype and chemotherapy were independent factors associated with the refusal of radiotherapy.

**Conclusions:**

Postoperative radiotherapy can improve the benefits of breast-conserving surgery. Patients with old age, low income, divorce, white race, advanced stage, and no chemotherapy were more likely to refuse radiotherapy.

**Supplementary Information:**

The online version contains supplementary material available at 10.1186/s13014-023-02297-2.

## Background

Breast cancer has become the malignant tumor with the highest incidence [1]. Although breast cancer has the highest incidence rate, its mortality rate had been decreasing, with a 43% decline between 1989 and 2020, and it was concentrated in larger regions [2].

The decline in breast cancer mortality was mainly due to the standardized treatment of breast cancer. The earliest classical treatment for breast cancer was radical mastectomy proposed by Halsted in 1894. This procedure greatly reduced the mortality and recurrence of breast cancer. But it also brought great side effects. In order to reduce the side effects caused by extended radical surgery, breast cancer surgery had been gradually changed from radical mastectomy to modified radical mastectomy and total mastectomy. However, breast deficiency causes great physical and psychological trauma in patients. With the advent of multidisciplinary diagnosis and treatment of cancer, surgical methods of breast cancer have begun to show a trend of "subtraction". With the development of radiotherapy, breast-conserving surgery has become the standard treatment for early breast cancer [3, 4]. With the development of time, the proportion of breast-conserving surgery also increased. By 2018, 63% of early-stage patients had undergone breast-conserving surgery with or without adjuvant radiotherapy [2].

With the diagnosis and treatment of breast cancer becoming more and more standardized, the mortality of breast cancer patients after breast-conserving surgery also decreased. Radiotherapy after breast-conserving surgery has become the standard treatment for breast cancer patients [5, 6]. However, there were still some patients who did not receive radiotherapy after breast-conserving therapy. Studies have shown that the local control of patients treated with breast irradiation after breast-conserving therapy was about one-third that of patients treated with breast-conserving therapy alone, so radiotherapy is essential for breast-conserving therapy [7, 8]. Therefore, standardized radiotherapy after breast-conserving surgery is necessary. However, studies showed that about 1.5% of patients treated with breast-conserving therapy in Korea didn’t receive postoperative radiotherapy [9]. Even more surprising was the increase in the rate of radiotherapy omission for breast-conserving patients in the United States from 1992 (15.5%) to 2007 (25%) [10]. Refusal of radiotherapy after breast-conserving surgery exists in all regions, and there are differences.

Refusal of radiotherapy can lead to non-standard treatment of breast cancer. It increases local recurrence and mortality of breast cancer and increases medical costs in the later period. Therefore, in this study, we selected patients who refused radiotherapy to further explore the factors influencing their decision to refuse radiotherapy.

## Methods

### Data source and study population

Women with a diagnosis of non-metastatic breast cancer between January 1, 2010, and December 31, 2015 were identified in the Surveillance, Epidemiology, and End Results (SEER) database. The SEER program registry consists of population-based cancer registries with data that cover approximately 28% of the US population. Radiotherapy refusal was defined as those patients who were recommended radiotherapy but did not undergo it due to the discretionary choice of the patient. Patients with unknown radiotherapy status were excluded.

The inclusion criteria for this study were: (1) age > 18 years old; (2) breast-conserving surgery was performed; (3) tumor origin of breast cancer; (4) the diagnosis time was from 2010 to 2015; (5) Only patients who refused or received radiotherapy were included in this analysis. Exclusion criteria: (1) diagnosis was confirmed by biopsy or autopsy only; (2) survival less than 1 month; (3) important clinicopathological information was missing; (4) the clinical stage was stage IV.

The use of a public database for this study was considered exempt from institutional review board approval.

### Statistical analysis

To investigate differences in patients' radiotherapy decisions, the study cohort was divided into two treatment statuses: receiving radiotherapy and refusal radiotherapy. Breast cancer specific survival (BCSS) was defined as the time from the date of diagnosis to the date of death from breast cancer. Overall survival (OS) was defined as the time from the date of diagnosis to death from any cause or the last follow-up. Sociodemographic and clinical variables were compared between groups, including age, median resident income, marital status, race, grade, clinical stage, T stage, N stage, estrogen receptor (ER) status, progesterone receptor (PR) status, human epidermal growth factor receptor (HER-2) status, molecular subtype, and chemotherapy. Sociodemographic and clinical variables were evaluated as classified variables. The Pearson chi-square test was used to analyze categorical variables between groups. Kaplan–Meier curves and log-rank tests were used to calculate survival for eligible patients with radiotherapy decision (radiotherapy versus radiotherapy refusal). To eliminate baseline differences between the two groups, the propensity score matching (PSM) was used to match the two groups for age, race, marital status, median resident income, ER, PR, HER-2, and chemotherapy. Multivariate Cox proportional hazards model was developed for eligible patients using patient clinicopathological factors. Multivariate logistic regression analysis was used to identify factors associated with the radiotherapy decision. A nomogram was established to predict radiotherapy refusal after breast-conserving surgery using significant variables in multivariate logistic regression.

The values of *p* < 0.05 was considered statistically significant. Statistical analyzes for this study were performed using IBM SPSS Statistics (version 22.0) and R software (version 4.0.3).

## Results

### Description of the study population

The study included 87,100 patients who underwent breast-conserving surgery for breast cancer between 2010 and 2015. Of all participants, 84,948 women (97.5%) received radiotherapy. 2152 women (2.5%) refused radiotherapy. Compared with the radiotherapy performed group, the patients who refused radiotherapy were older, had lower income, and a greater proportion were white. The patients who refused radiotherapy had a lower grade, earlier clinical stage, lower N stage, higher rates of ER-positive and higher rates of PR-positive. The proportion of patients who received chemotherapy was lower among those who refused radiotherapy (Table 1).

**Table 1:** Proportion of patients with breast conserving surgery who underwent radiotherapy and those who refused radiotherapy diagnosed between 2010 and 2015

	Radiotherapy performed		Radiotherapy refused		*P*-value
	No	%	No	%	
*Years at diagnosis*					
2010	12,247	14.40	261	12.10	< 0.001
2011	13,254	15.60	283	13.20	
2012	13,577	16.00	359	16.70	
2013	14,048	16.50	321	14.90	
2014	15,284	18.00	391	18.20	
2015	16,538	19.50	537	25.00	
*Age*					
≤ 50	17,165	20.20	211	9.80	< 0.001
> 50	67,783	79.80	1941	90.20	
*Income*					
< 65,000	40,684	47.90	1328	61.70	< 0.001
≥ 65,000	44,264	52.10	824	38.30	
*Marital*					
Married	52,983	62.40	974	45.30	< 0.001
Single	12,068	14.20	291	13.50	
Divorced	19,897	23.40	887	41.20	
*Race*					
White	69,215	81.50	1860	86.40	< 0.001
Black	7855	9.20	170	7.90	
Others	7878	9.30	122	5.70	
*Grade*					
Grade I + II	62,435	73.50	1660	77.10	< 0.001
Grade III + IV	22,513	26.50	492	22.90	
*Stage*					
Stage I	54,636	64.30	1479	68.70	< 0.001
Stage II	27,056	31.90	605	28.10	
Stage III	3256	3.80	68	3.20	
*T stage*					
T1	63,396	74.60	1611	74.90	< 0.001
T2	19,922	23.50	491	22.80	
T3	1335	1.60	29	1.30	
T4	295	0.30	21	1.00	
*N stage*					
N0	67,975	80.00	1870	86.90	< 0.001
N1	14,419	17.00	238	11.10	
N2	1893	2.20	29	1.30	
N3	661	0.80	15	0.70	
*ER status*					
Negative	11,098	13.10	208	9.70	< 0.001
Positive	73,850	86.90	1944	90.30	
*PR status*					
Negative	18,823	22.20	396	18.40	< 0.001
Positive	66,125	77.80	1756	81.60	
*HER-2 status*					
Negative	75,448	88.80	1955	90.80	0.003
Positive	9500	11.20	197	9.20	
*Subtype*					
HR + /HER-2 +	7048	8.30	148	6.90	< 0.001
HR + /HER-2-	67,543	79.50	1809	84.10	
HR-/HER-2 +	2452	2.90	49	2.30	
HR-/HER-2-	7905	9.30	146	6.80	
*Chemotherapy*					
No	55,663	65.50	1964	91.30	< 0.001
Yes	29,285	34.50	188	8.70	

**Fig. 1:**
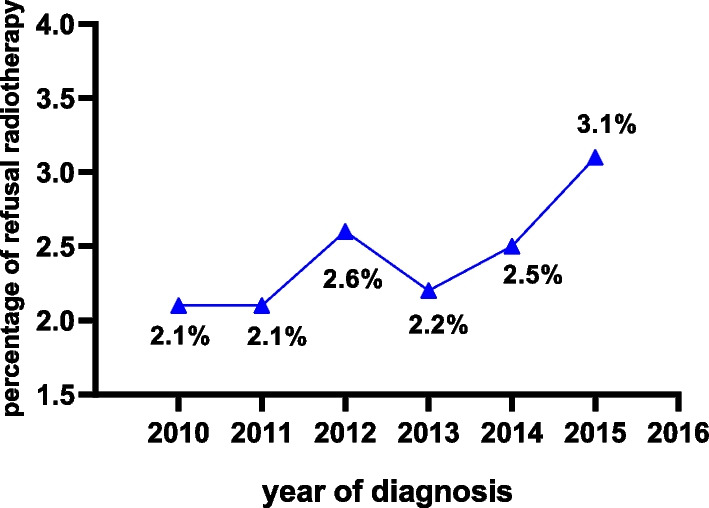
Proportion of breast cancer patients refusing radiotherapy after breast-conserving surgery diagnosed between 2010 and 2015

The rate of refusal of radiotherapy after breast-conserving surgery increased significantly during the study period as the time of diagnosis moved later, from 2.1% in 2010 to 3.1% in 2015 (Fig. 1).

### Survival analysis of breast conserving patients treated with radiotherapy

**Fig. 2:**
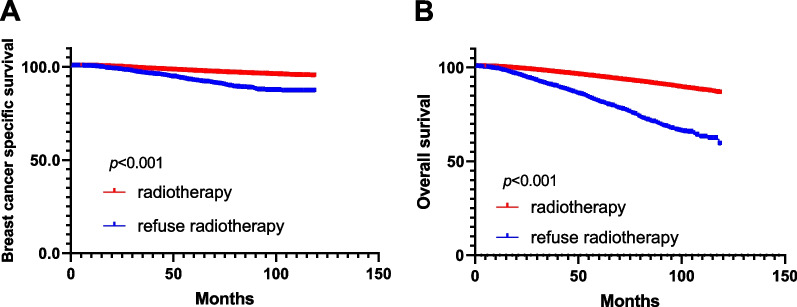
Kaplan–Meier survival curves of overall survival and breast cancer-specific survival stratified by radiotherapy and refusal radiotherapy (**A**: BCSS;** B**: OS)

The Kaplan–Meier survival curve showed that OS and BCSS of breast cancer patients who received radiotherapy were better than those who refused radiotherapy (Fig. 2, *p* < 0.001). Similarly, in the subgroup analysis, we found that radiotherapy improved OS in patients with breast conserving surgery in age ≤ 50 and age > 50 (Figure S1 A, B, all *p* < 0.001). In clinical stage subgroup analysis, radiotherapy improve OS of patients with breast conserving surgery, with statistically significant differences (Additional file 1: Fig. S1C, D, E, all *p* < 0.001). Similarly, radiotherapy improve OS of four molecular subtypes of breast cancer (hormone receptor/HER-2) (Additional file 1: Fig. S1 F, G, H and I, all *p* < 0.001).

For patients receiving breast cancer conservation therapy, we included radiotherapy, age, income, marital status, race, grade, clinical stage, subtype, and chemotherapy as potential prognostic variables in the multivariate model. Multivariate cox regression analysis showed that refusing radiotherapy significantly decreased BCSS (HR 3.457, 95% CI (2.977–4.014), *p* < 0.001) and OS (HR 2.879, 95% CI (2.632–3.15),* p* < 0.001) (Table 2). Other factors including age, income, marital status, race, grade, clinical stage, subtype, and chemotherapy were identified as independent significant predictors of BCSS and OS for breast cancer with breast-conserving surgery.

**Table 2:** Multivariate model of breast cancer-specific mortality and overall mortality in patients with breast conserving surgery

	BCSS		OS	
	HR (95% CI)	*P*-value	HR (95% CI)	*P*-value
Radiotherapy		< 0.001		< 0.001
Perform	Reference		Reference	
Refused	3.457 (2.977–4.014)	< 0.001	2.879 (2.632–3.15)	< 0.001
Age		< 0.001		< 0.001
≤ 50	Reference		Reference	
> 50	1.19 (1.092–1.296)	< 0.001	1.806 (1.682–1.938)	< 0.001
Income		< 0.001		< 0.001
< 65,000	Reference		Reference	
≥ 65,000	0.855 (0.796–0.918)	< 0.001	0.864 (0.826–0.905)	< 0.001
Marital		< 0.001		< 0.001
Married	Reference		Reference	
Single	1.177 (1.068–1.298)	0.001	1.287 (1.201–1.379)	< 0.001
Divorced	1.389 (1.281–1.505)	< 0.001	1.915 (1.822–2.013)	< 0.001
Race		< 0.001		< 0.001
White	Reference		Reference	
Black	1.274 (1.158–1.402)	< 0.001	1.141 (1.064–1.223)	< 0.001
Others	0.78 (0.676–0.901)	0.001	0.728 (0.66–0.803)	< 0.001
Grade		< 0.001		< 0.001
Grade I + II	Reference		Reference	
Grade III + IV	2.542 (2.331–2.771)	< 0.001	1.717 (1.622–1.818)	< 0.001
Stage		< 0.001		< 0.001
Stage I	Reference		Reference	
Stage II	3.172 (2.905–3.464)	< 0.001	2.061 (1.958–2.169)	< 0.001
Stage III	9.868 (8.812–11.052)	< 0.001	5.417 (4.981–5.891)	< 0.001
Subtype		< 0.001		< 0.001
HR + /HER-2 +	Reference		Reference	
HR + /HER-2-	1.476 (1.284–1.697)	< 0.001	1.127 (1.025–1.24)	0.013
HR-/HER-2 +	1.576 (1.294–1.919)	< 0.001	1.301 (1.122–1.509)	< 0.001
HR-/HER-2-	2.87 (2.487–3.312)	< 0.001	2.08 (1.877–2.304)	< 0.001
Chemotherapy		0.008		< 0.001
No	Reference		Reference	
Yes	1.136 (1.034–1.247)	0.008	0.601 (0.565–0.639)	< 0.001

### After PSM, radiotherapy was used as a prognostic factor for survival

**Fig. 3:**
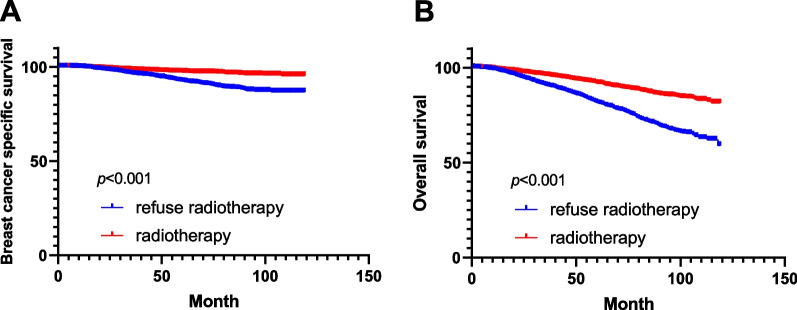
Kaplan–Meier survival curves of breast cancer-specific survival and overall survival stratified by radiotherapy and refusal radiotherapy after PSM

To further confirm the results of the multivariate proportional hazards regression, we performed PSM to adjustment analysis. A total of 2144 patients who received radiotherapy were matched to 2144 patients who refused radiotherapy. After matching, the variables in age (*p* = 0.375), income (*p* = 0.875), marital status (*p* = 0.253), race (*p* = 0.826), grade (*p* = 0.081), clinical stage (*p* = 0.363), T stage (*p* = 0.813), N stage (*p* = 0.766), ER status (*p* = 0.714), PR status (*p* = 0.783) and HER-2 status (*p* = 0.274), subtype (*p* = 0.749) and chemotherapy (*p* = 1) were not statistically different between the two groups. (Table 3). Using Kaplan–Meier survival analysis, radiotherapy improved BCSS (*p* < 0.001) in the post-matched cohort (Fig. 3A). The OS of patients who also received radiotherapy was higher than that of those who refused radiotherapy in the post-matched cohort (*p* < 0.001) (Fig. 3B).

**Table 3:** Comparisons of clinicopathological characteristics between the radiotherapy perform and radiotherapy refused group in 1:1 matched case–control analysis

	Radiotherapy performed		Radiotherapy refused		*P*-value
	No	%	No	%	
*Age*					
≤ 50	194	9.00	211	9.80	0.375
> 50	1950	91.00	1933	90.20	
*Income*					
< 65,000	1325	61.80	1320	61.60	0.875
≥ 65,000	819	38.20	824	38.40	
*Marital*					
Married	948	44.20	972	45.30	0.253
Single	266	12.40	291	13.60	
Divorced	930	43.40	881	41.10	
*Race*					
White	1839	85.80	1853	86.40	0.826
Black	178	8.30	170	7.90	
Others	127	5.90	121	5.60	
*Grade*					
Grade I + II	1705	79.50	1658	77.30	0.081
Grade III + IV	439	20.50	486	22.70	
*Stage*					
Stage I	1516	70.70	1479	69.00	0.363
Stage II	562	26.20	603	28.10	
Stage III	66	3.10	62	2.90	
*T stage*					
T1	1637	76.40	1610	75.10	0.813
T2	463	21.60	489	22.80	
T3	27	1.30	28	1.30	
T4	17	0.80	17	0.80	
*N stage*					
N0	1872	87.30	1866	87.00	0.766
N1	225	10.50	237	11.10	
N2	29	1.40	28	1.30	
N3	18	0.80	13	0.60	
*ER status*					
Negative	197	9.20	204	9.50	0.714
Positive	1947	90.80	1940	90.50	
*PR status*					
Negative	398	18.60	391	18.20	0.783
Positive	1746	81.40	1753	81.80	
*HER-2 status*					
Negative	1971	91.90	1951	91.00	0.274
Positive	173	8.10	193	9.00	
*Subtype*					
HR + /HER-2 +	130	6.10	146	6.80	0.749
HR + /HER-2-	1827	85.20	1807	84.30	
HR-/HER-2 +	43	2.00	47	2.20	
HR-/HER-2-	144	6.70	144	6.70	
*Chemotherapy*					
No	1956	91.20	1956	91.20	1.0
Yes	188	8.80	188	8.80

### Predictors of radiotherapy refusal after breast-conserving surgery

**Fig. 4:**
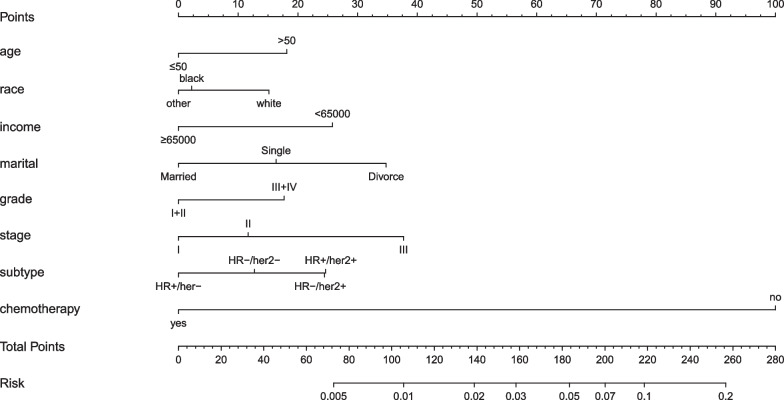
Nomogram predicted refusal radiotherapy for patients with breast conserving surgery

The results of the multivariate logistic regression were shown in Table 4. The results confirmed that age, income, marital status, race, grade, stage, molecular subtype and chemotherapy were independent factors associated with the refusal of radiotherapy. Patients with older, low income, divorce, white race, high grade, advanced stage, and no chemotherapy were more likely to refuse radiotherapy. A nomogram was developed using R software to predict the refusal of radiotherapy on the basis of independent factors for the refusal of radiotherapy (Fig. 4). From the Fig. 4, we can conclude that HR-/HER-2+ and HR+/HER-2+ patients had a higher tendency to refuse radiotherapy. Among them, chemotherapy had the largest weight in predicting the refusal of radiotherapy. The absence of chemotherapy significantly increased the probability that patients would refuse radiotherapy.

**Table 4:** Multivariate logistic regressions model for predictors of radiotherapy refused

Factor	OR	95%CI	*P*-value
Age			< 0.001
≤ 50	1	Reference	
> 50	1.470	1.269–1.703	< 0.001
Income			< 0.001
< 65,000	1	Reference	
≥ 65,000	0.578	0.529–0.632	< 0.001
Marital			< 0.001
Married	1	Reference	
Single	1.415	1.237–1.619	< 0.001
Divorced	2.094	1.905–2.301	< 0.001
Race			< 0.001
White	1	Reference	
Black	0.759	0.645–0.894	0.001
Others	0.725	0.601–0.874	0.001
Grade			< 0.001
Grade I + II	1	Reference	
Grade III + IV	1.457	1.293–1.642	< 0.001
Stage			< 0.001
Stage I	1	Reference	
Stage II	1.281	1.159–1.416	< 0.001
Stage III	2.228	1.712–2.898	< 0.001
Subtype			< 0.001
HR + /HER-2 +	1	Reference	
HR + /HER-2-	0.593	0.495–0.71	< 0.001
HR-/HER-2 +	0.997	0.713–1.392	0.984
HR-/HER-2-	0.776	0.612–0.985	0.037
Chemotherapy			< 0.001
No	1	Reference	
Yes	0.119	0.1–0.142	< 0.001

## Discussion

With the development of new chemotherapy drugs, targeted drugs, and radiation technology, the treatment effect of breast cancer is getting better and better. It should be noted that in the Cox regression of BCSS in breast-conserving patients, our study showed that chemotherapy would improve the survival risk of patients. In our subsequent analysis, we found that this was due to the baseline difference between the groups receiving chemotherapy and those not receiving chemotherapy. Patients who received chemotherapy typically had a higher tumor burden and a later clinical stage. The meta-analysis of EBCTCG showed that radiotherapy after breast-conserving surgery can reduce recurrence and mortality compared with no radiotherapy (median follow-up 9.5 years) [4]. Radiotherapy after breast-conserving surgery for breast cancer is also the standard mode recommended by various guidelines around the world [11, 12]. However, it should be noted that the proportion of patients refusing radiotherapy after breast-conserving surgery increased from 2.1% in 2010 to 3.1% in 2015. Our study also showed that radiotherapy could improve OS and BCSS in patients with breast-conserving surgery. After baseline differences were eliminated using PSM, our study similarly suggested that radiotherapy could improve OS and BCSS in breast-conserving patients. Therefore, the benefit of radiotherapy in breast-conserving patients with breast cancer is unanimously affirmed.

The compliance with treatment of breast cancer patients is affected by many aspects. It includes the physician, the disease, and the patient [13, 14]. To standardize the diagnosis and treatment of breast cancer patients, it can be strengthened from the above three main aspects.

As a chronic disease, breast cancer belongs to the “mutual participation mode” in the relationship between doctors and patients [15]. The mutual participation model is based on a relationship in which doctors and patients work together, assume shared responsibility, and make shared decisions [16]. As the doctor in charge of breast cancer treatment, he plays an important role in patient compliance. The treatment sequence of breast cancer patients with breast-conserving surgery is usually chemotherapy first, followed by radiotherapy after recovery from breast-conserving surgery. If there was no indication for chemotherapy, radiotherapy was performed directly [17]. Observation by the physician in time and postoperative follow-up are particularly important. Similarly, the psychology of breast cancer patients is generally more anxious, and timely intervention of psychological treatment can reduce patient anxiety and improve patient health. Therefore, the timely intervention of psychological team also plays an important role in today's multidisciplinary treatment (MDT). The variables in this study do not include the physician’s aspect, but we cannot ignore the role that the physician plays in the overall disease process. Therefore, more prospective clinical studies that take the physician aspect into account in the future are necessary.

In terms of disease, our study showed that patients with high grade, advanced clinical stage, and absence of chemotherapy have a lower incidence of radiotherapy after breast-conserving surgery. However, this study has excluded stage IV breast cancer, and most early breast cancers are curable. Therefore, for patients undergoing breast-conserving therapy, if their tumor grade and stage are later, we should be alert that patients will give up standardized radiotherapy. In our study, chemotherapy had the highest weight in predicting rejection of radiotherapy (Fig. 4). Patients who did not receive chemotherapy were more likely to refuse radiotherapy. In this study, breast-conserving patients did not receive chemotherapy, which may be due to two reasons. One is that some breast-conserving patients had early disease and their condition did not require chemotherapy. Another is partly due to patients refusing chemotherapy for various reasons. Therefore, physicians should also conduct a good follow-up of early patients who are exempt from chemotherapy to ensure that patients receive subsequent radiotherapy.

In terms of patients, older, low income, divorce, and white race were risk factors for refusing radiotherapy after breast-conserving surgery. Older patients are generally associated with more heart diseases, such as hypertension and coronary heart disease. Radiotherapy may increase the risk of early radiation-induced heart damage and increase the risk of radiation-related heart disease death, especially when the tumor is located on the left side [18]. Therefore, for patients with left breast cancer, advanced radiotherapy technology can be used to protect heart function [19, 20]. With the advent of hypofractionated radiotherapy, the efficacy and side effects of radiotherapy are not significantly different from those of conventional radiotherapy, but also greatly shorten the treatment time of patients and increase the compliance of patients [21]. Similarly, low income and divorced people need social assistance. It is important to note that white people were also a risk factor for radiotherapy refusal in our study. For stage III breast cancer, black women were more likely to receive radiation or chemotherapy alone [22]. Studies have shown that doctors are biased against patients' race. Although many physicians, regardless of specialty, show an implicit preference for whites, this bias does not appear to affect their clinical decisions [23]. Thus, we need better follow-up of white patients when race is known.

Although our study obtained the influencing factors of radiotherapy refusal in breast-conserving patients and constructed a model to predict the refusal of radiotherapy after breast-conserving surgery. However, the study also has several limitations. First, this study is a retrospective study. There are biases that are inevitable in retrospective studies, such as selection bias. Second, important information, such as Ki-67, chemotherapy regimen, and radiotherapy regimen could not be obtained in this study. Finally, the model we developed was not externally validated. Therefore, more prospective multicenter studies are needed to explorer in the further.

## Conclusions

In the treatment of breast cancer, radiotherapy can improve the benefits of breast conserving surgery. Patients with older, low income, divorce, white race, advanced stage, and no chemotherapy were more likely to refuse radiotherapy.

### Supplementary Information

**Additional file 1.** Kaplan–Meier OS curves for patients with breast conserving surgery according to different subgroups. A-I Kaplan–Meier OS curves for patients with breast conserving surgery according to A age ≤ 50, B age ＞ 50, C stage I, D stage II, E stage III, F HR+/HER-2+, G HR+/HER-2-, H HR-/HER-2+ and I HR-/HER-2-

## Data Availability

These data were publicly available for use in accordance with a limited use agreement for SEER research data: Surveillance, Epidemiology, and End Results (SEER) Program (https://seer.cancer.gov) SEER*Stat Database.

## Abbreviations

BCSS: Breast cancer specific survival
OS: Overall survival
ER: Estrogen receptor
PR: Progesterone receptor
HER-2: Human epidermal growth factor receptor 2
