# Supplementary figures and images for: Radiotherapy refusal in breast cancer with breast-conserving surgery
Source: Radiat Oncol. 2023 Aug 5;18:130. doi: 10.1186/s13014-023-02297-2 (PMC10403910; doi:10.1186/s13014-023-02297-2)

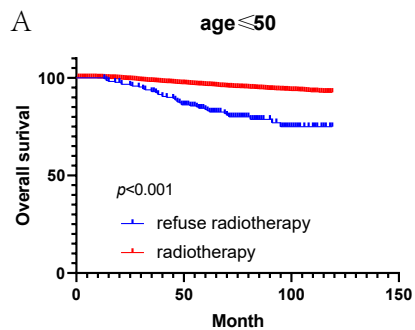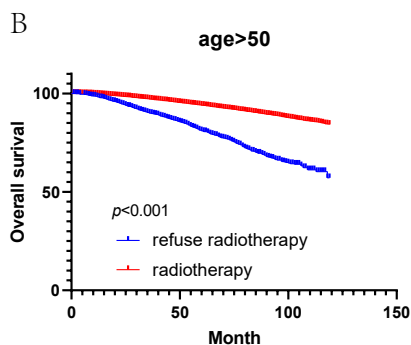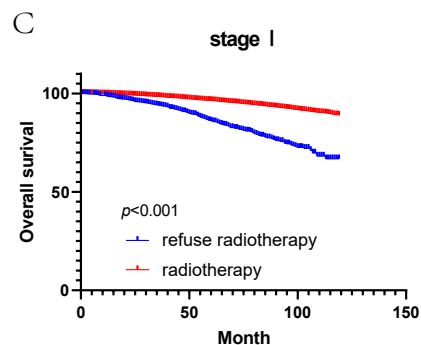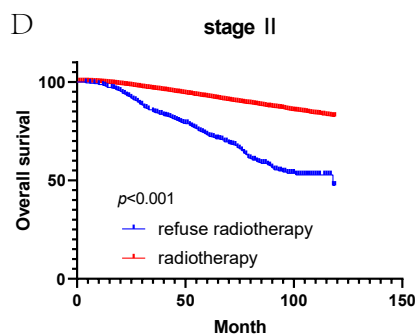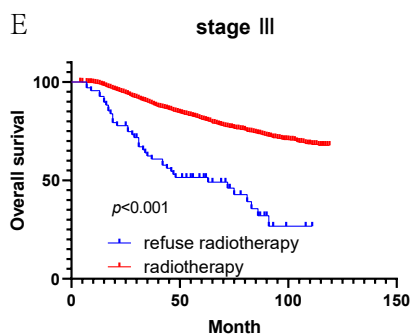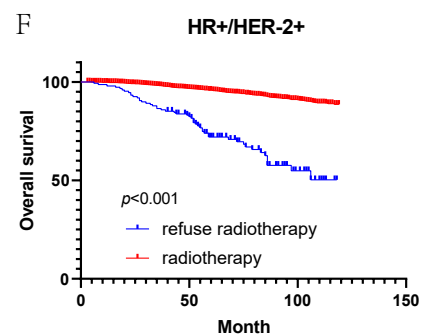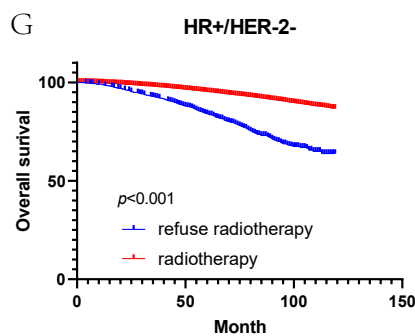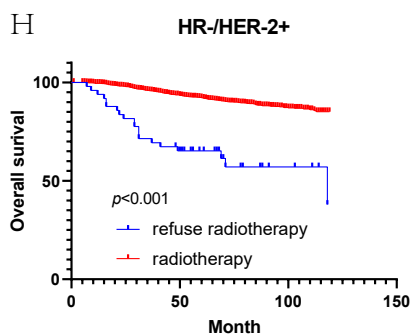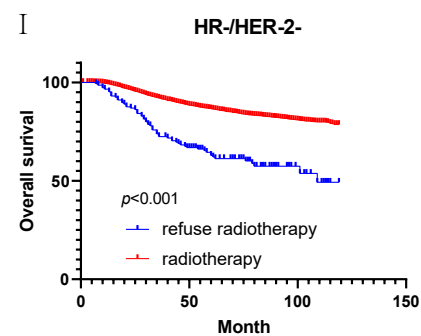

Supplement: Supplementary file 1 — Additional file 1. Kaplan–Meier OS curves for patients with breast conserving surgery according to different subgroups. A-I Kaplan–Meier OS curves for patients with breast conserving surgery according to A age ≤ 50, B age ＞ 50, C stage I, D stage II, E stage III, F HR+/HER-2+, G HR+/HER-2-, H HR-/HER-2+ and I HR-/HER-2- [file 13014_2023_2297_MOESM1_ESM.pdf]
